# Urinary tract infection with *Corynebacterium aurimucosum* after urethroplasty stricture of the urethra: a case report

**DOI:** 10.1186/s13256-015-0638-0

**Published:** 2015-07-14

**Authors:** Seynabou Lo, Issa Thiam, Bécaye Fall, Awa Ba-Diallo, Oumarou Foly Diallo, Rokhaya Diagne, Mamadou Lamine Dia, Roughyatou Ka, Aicha Marceline Sarr, Ahmad Iyane Sow

**Affiliations:** 1 Faculty of Health Sciences, Gaston Berger University of Saint Louis, PO Box: 234, Saint Louis, Senegal; 2 Regional Hospital Center of Saint Louis, PO Box: 401, Saint Louis, Senegal; 3 Hôpital Principal de Dakar, 1, avenue Nelson Mandela, PO Box: 3006 Dakar, Senegal; 4 Faculty of Medicine Pharmacy and Odonto-Stomatology, Cheikh Anta Diop University, PO Box: 22254, Dakar-Ponty, Senegal; 5 Laboratories Directorate of Senegal, Ministry of Health and Prevention, Sacré Coeur 3, Pyrotechnie, Dakar, Senegal

**Keywords:** *Corynebacterium aurimucosum*, Mass spectrometry, Urethroplasty

## Abstract

**Introduction:**

*Corynebacteria* have an important place among the commensal flora of the skin and mucous membranes. Except for *Corynebacterium diphtheriae*, they were once considered contaminants of mucosa. Recent publications in medical bacteriology have highlighted the importance of several species, such as *C. aurimucosum*. To the best of our knowledge, we report the first isolation of this strain from urine.

**Case presentation:**

We report a case of a patient with a urinary tract infection with *C. aurimucosum. We* isolated this bacterium from a 52-year-old man of Wolof ethniticity (an ethnic group in Senegal, West Africa) at the regional hospital of Saint Louis, Senegal. Microscopic examination of his total urine sample showed coryneform Gram-positive bacilli associated with a high leukocyte reaction. After repeated isolation of the corynebacteria in three samples from the patient’s urine, it was identified by matrix-assisted laser desorption/ionization time-of-flight mass spectrometry. The strain was susceptible to antibiotics, except for penicillin and co-trimoxazole. The potential infectious role of these commensal species in several infections should be taken into consideration.

**Conclusions:**

This case highlights the significant proportion of species in the genus Corynebacterium other than dyphteriae in the infectious process. The use of mass spectrometry for identification highlights the originality of this work and the importance of these new diagnostic tools that are unavailable in most health facilities of countries with limited resources. We share the results of our method of identification of the isolated bacteria. This case should prompt attention to these rare bacteria, which can cause severe infections.

## Introduction

*Corynebacteria* are an important part of the commensal flora of the skin and mucous membranes. Except for *Corynebacterium diphtheriae*, they were once considered contaminants of mucosa [1, 2]. *Corynebacteria* can be found in many secretions from the oropharynx and in pus, skin ulcers and eye infections. Their form is either a slight stick shape or slightly curved, tapered at the ends or club-shaped, and they are often arranged in a V formation or as a parallel fence. They are non-motile, facultative aerobes or anaerobes, and they are catalase positive [3]. The pathology of these bacteria is still dominated by diphtheria, but recent publications in medical bacteriology have highlighted the importance of others, such as *C. amycolatum*, *C. jeikeium, C. urealyticum and C. seminale* [4].

## Case presentation

In this case report, we describe a patient with a urinary tract infection with *C. aurimucosum* who was treated at a regional hospital in Saint Louis, Senegal. The patient was a 52-year-old man of Wolof ethnicity (an ethnic group in Senegal, West Africa). He was a farmer (plants), polygamous (two wives) and lived in rural areas. He was received for consultation with a dysuric type of low voiding jet with thrust forces associated with urinary frequency during the night and hematuria.

His physical examination revealed pale mucous and alteration of his general condition. A wound with welling pus was observed under his testicles, and we noticed a scar shaped like an inverted “U” next to the perineal incision, probably related to a history of urethroplasty. The patient had undergone two previous urethroplasties, in 2002 and 2010. His prostate was found to be normal during a rectal examination, but echography showed a slight increase of the volume to 34.98ml. In the medical and surgical history of the patient, urine and pus examinations showed the presence of *Escherichia coli,* a member of a Gram-negative enterobacterial group. This infection was treated with ceftriaxone for 10 days, and it was completely cured; the result of his post-treatment urine culture was negative. Retrograde urethrocystography was done, which showed bulbar urethral stenosis associated with extravasation of the contrast fluid in the perineum (caused by the presence of a urethral cutaneous fistula). Urethroplasty was indicated and performed. In the post-operative stage, nothing out of the ordinary was observed, and the patient was released after a 10-day hospitalization. During the patient’s post-operative follow-up, we noticed repeated dilations with Benique’s probe (metal urethral probe). A urine control examination revealed the presence of Gram-positive coryneform bacilli associated with a high leukocyte reaction with crystals of phosphate, ammonia and magnesium (which can be caused by drugs or food debris).

**Fig. 1:**
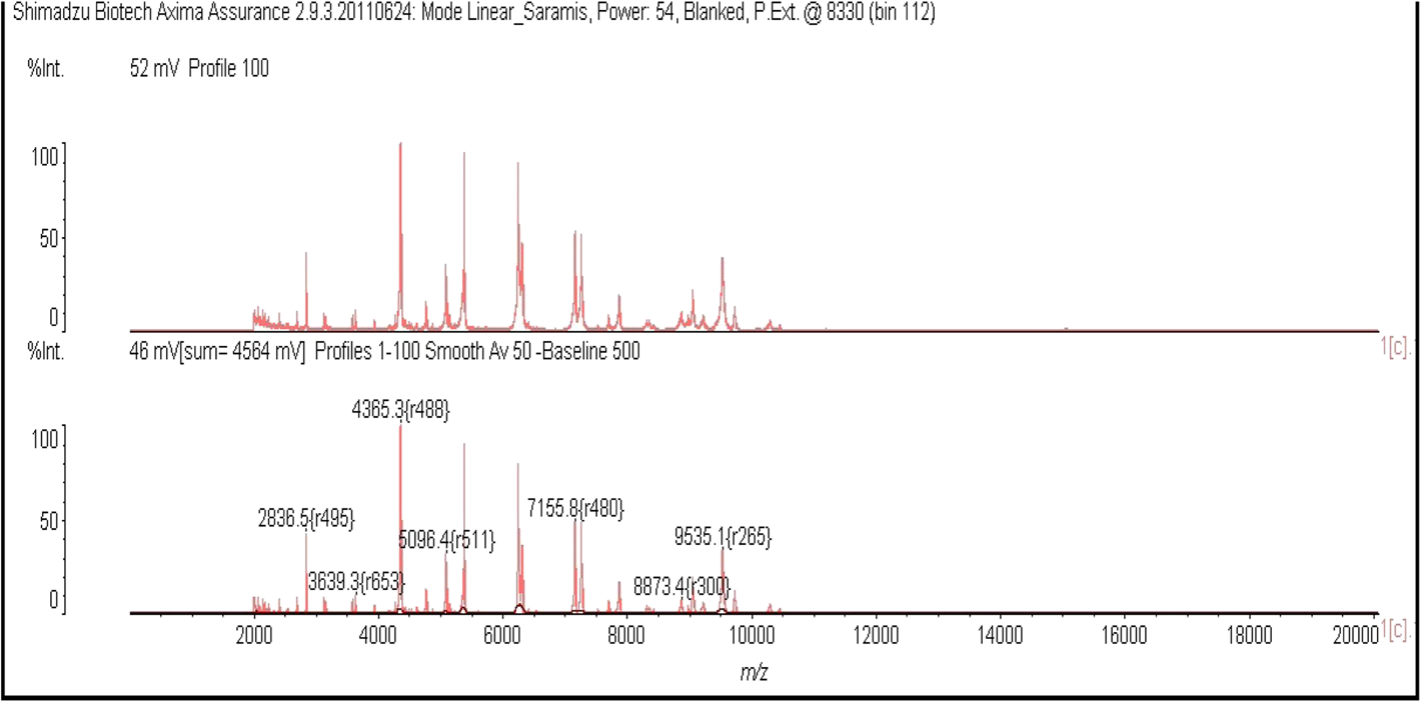
Mass spectral profile

A culture was done on cysteine-lactose electrolyte-deficient agar according to laboratory procedure. The result was a monomicrobial culture above 10^7^ colony-forming units per milliliter. A control sample was requested to ensure that there was no contamination. The repeated isolation of bacteria in three different urine samples confirmed the potential involvement of the bacteria in an infection. On ordinary blood agar, we noted grayish colonies of medium size without hemolysis. Among the biochemical items we studied, catalase was positive, but urease was negative. Matrix-assisted laser desorption/ionization time-of-flight (MALDI-TOF) mass spectrometry revealed the existence of *C. aurimucosum* (Fig. 1)*.* Anti-microbial susceptibility was performed by using a diffusion technique on ordinary blood agar according to the recommendations of the Antibiogram Committee of the French Society for Microbiology [5]. The strain was susceptible to erythromycin (15μg), pristinamycin (15μg), lincomycin (15μg), vancomycin (30μg), cefalothin (30μg), chloramphenicol (30μg), imipenem (30μg), tetracycline (30μg) and ciprofloxacin (30μg). However, resistance was observed with penicillin (6μg) and trimethoprim with sulfamethoxazole (1.25μg and 23.75μg, respectively). Treatment with imipenem 500mg twice daily for 10 days ended the infection. Improvement of the patient’s condition after treatment was recorded, and his outcome was favorable with no adverse effects.

## Discussion

*The Escherichia coli* is a frequently encountered type of enterobacteria isolated in urine. In a study conducted in our hospital, it represented up to 50% of all urinary Enterobacteriaceae isolates [6]. In our patient, the contrast fluid in the perineum revealed the presence of a urethral cutaneous fistula, which explained the possible origin of *Escherichia coli enterobacteria. C. aurimucosum* was isolated after the patient underwent urethroplasty. This strain is often isolated in several clinical samples; it can cause a skin affection or erythrasma [7]. According to several studies, *C. aurimucosum* and other species *C. amycolatum*, *C. jeikeium* and *C. striatum* belong to the main species of corynebacteria occasionally responsible for prosthetic joint infection [1]. *C. aurimucosum* was isolated in vaginal secretions of a 34-year-old woman who had undergone an abortion in the sixth month of pregnancy, and its pathogenic role was demonstrated [8]. This bacterium was found to account for 13.6% of non-diphtheria corynebacteria identified by using molecular techniques in a French population [9]. On the phylogenetic and molecular levels, *C. aurimucosum* has been characterized and its genome completely sequenced [10]. Regarding sensitivity to antibiotics, our patient was resistant only to penicillin and trimethoprim with sulfamethoxazole. A study on species of corynebacteria showed resistance up to 50% to penicillin, erythromycin and clindamycin [11]. Our literature review showed that this kind of *Corynebacterium* has never been isolated in urine. Our study shows its potential infectious role. In addition, the new techniques of bacteria identification that we used do not exist in most of our health facilities of countries with limited resources.

## Conclusions

This case demonstrates the importance of bacterial identification in some unusual urinary infections caused by commensal bacteria, which can be responsible for severe infections. *C. aurimucosum* has never been isolated in urine, and therefore the importance of new identification techniques such as MALDI-TOF mass spectrometry, which does not exist in our laboratory where we are often faced with problems concerning the identification of rare bacteria.

## Consent

Written informed consent was obtained from the patient for publication of this case report and any accompanying images. A copy of the written consent is available for review by the Editor-in-Chief of this journal.

## Abbreviation

MALDI-TOF: Matrix-assisted laser desorption/ionization time-of-flight
